# Oral chromium picolinate impedes hyperglycemia-induced atherosclerosis and inhibits proatherogenic protein TSP-1 expression in STZ-induced type 1 diabetic ApoE^−/−^ mice

**DOI:** 10.1038/srep45279

**Published:** 2017-03-27

**Authors:** Rituparna Ganguly, Soumyadip Sahu, Vahagn Ohanyan, Rebecca Haney, Ronaldo J. Chavez, Shivani Shah, Siri Yalamanchili, Priya Raman

**Affiliations:** 1 Department of Integrative Medical Sciences, Northeast Ohio Medical University, 4209 State Route 44, Rootstown, OH 44272, USA; 2 School of Biomedical Sciences, Kent State University, Kent, OH 44240, USA

## Abstract

Increasing evidence suggests thrombospondin-1 (TSP-1), a potent proatherogenic matricellular protein, as a putative link between hyperglycemia and atherosclerotic complications in diabetes. We previously reported that the micronutrient chromium picolinate (CrP), with long-standing cardiovascular benefits, inhibits TSP-1 expression in glucose-stimulated human aortic smooth muscle cells *in vitro*. Here, we investigated the atheroprotective action of orally administered CrP in type 1 diabetic apolipoprotein E-deficient (ApoE^−/−^) mice and elucidated the role of TSP-1 in this process. CrP decreased lipid burden and neointimal thickness in aortic root lesions of hyperglycemic ApoE^−/−^ mice; also, smooth muscle cell (SMC), macrophage and leukocyte abundance was prevented coupled with reduced cell proliferation. Attenuated lesion progression was accompanied with inhibition of hyperglycemia-induced TSP-1 expression and reduced protein O-glycosylation following CrP treatment; also, PCNA and vimentin (SMC synthetic marker) expression were reduced while SM-MHC (SMC contractile marker) levels were increased. To confirm a direct role of TSP-1 in diabetic atherosclerosis, hyperglycemic TSP-1^−/−^/ApoE^−/−^ double knockout mice were compared with age-matched hyperglycemic ApoE^−/−^ littermates. Lack of TSP-1 prevented lesion formation in hyperglycemic ApoE^−/−^ mice, mimicking the atheroprotective phenotype of CrP-treated mice. These results suggest that therapeutic TSP-1 inhibition may have important atheroprotective potential in diabetic vascular disease.

Vascular disease is the leading cause of increased morbidity and mortality in diabetes. Risks of atherosclerotic complications are enhanced two-to-four-fold in diabetic patients^1^,^2^, accounting for >80% of deaths and hospitalizations in these individuals. Clinical studies, including recent animal data^3^,^4^,^5^,^6^,^7^, indicate that elevated glucose levels, independent of hyperlipidemia, may have profound proatherogenic effects in diabetes. Previous epidemiological studies^1^,^2^ have revealed a strong association between cumulative glycemic exposure and intima-media thickness (IMT) of the carotid artery, an early marker of atherosclerosis. Together, these reports support the notion that hyperglycemia is an important risk factor for development of macrovascular complications in diabetes. However, mechanisms underlying hyperglycemia-induced atherosclerosis are incompletely understood.

Thrombospondin-1 (TSP-1), a potent proatherogenic and anti-angiogenic protein, belongs to a family of matricellular proteins controlling cell-cell and cell-matrix interactions^8^. Earlier studies have shown that TSP-1 expression is significantly enhanced in response to vascular injury and in atherosclerotic lesions, with augmented expression in vascular smooth muscle cells (VSMC)^9^,^10^,^11^. Growing literature indicates distinct cell-and tissue-specific effects of TSP-1; both *in vivo* and *in vitro* studies have revealed that TSP-1 stimulates VSMC proliferation^12^ while inducing endothelial cell (EC) apoptosis^13^. The TSP protein family has been previously linked to atherosclerotic vascular disease based on GENEQUEST studies demonstrating an association between specific single nucleotide polymorphisms in the TSP genes with coronary artery disease and myocardial infarction^14^. These findings, confirmed by multiple human studies, lend support to TSP-1 as an alternative pathway for development of atherosclerosis.

Diabetic patients and diabetic animal models have been reported to have elevated TSP-1 levels in the plasma and walls of the large blood vessels^15^,^16^. Earlier work demonstrated that high glucose *in vitro*, characteristic of a diabetic milieu, upregulates TSP-1 expression in cells of the large blood vessel (VSMC, EC, fibroblasts)^16^. We have further shown that hyperglycemia *in vitro* increases TSP-1 expression via a transcriptional mechanism in primary human aortic smooth muscle cell (HASMC) cultures^17^,^18^. Together, these findings implicate TSP-1 as a putative link between hyperglycemia and accelerated atherosclerotic complications in diabetes. However, the impact of therapeutic TSP-1 inhibition on diabetic atherogenesis remains unexplored.

We recently reported^19^ that chromium picolinate (CrP), the most bioavailable form of the mineral nutrient trivalent chromium (Cr3+) at pharmacological concentrations, inhibits TSP-1 expression in glucose-stimulated HASMC *in vitro*. In addition, we have found that TSP-1 inhibition was accompanied with attenuated HASMC proliferation in response to Cr3+ and this effect was specific for high glucose conditions^19^. Accumulating data have indicated optimal regulatory effects of Cr3+ on carbohydrate and lipid metabolism^20^,^21^. Unlike its hexavalent counterpart (Cr6+), Cr3+ is relatively stable, with minimal toxicity issues at doses allowable for dietary intake^22^,^23^. Numerous studies have also indicated favorable glycemic and cardiovascular effects of Cr3+ 24,25,26,27,28. Nevertheless, clinical significance of Cr3+ in health and disease has been challenged by a dearth of mechanistic understanding of Cr3+ action.

Diabetic patients have low circulating and tissue Cr3+ levels compared to non-diabetic individuals^29^. There is increasing evidence that inadequate Cr3+ intake may elevate blood glucose and lipid levels^30^. Clinical studies have revealed that CrP in combination with biotin reduces insulin resistance and lowers the plasma atherogenic index in a cohort of type 2 diabetic patients^31^. Previous studies have also demonstrated that in STZ-induced diabetic Sprague Dawley rats *in vivo* and glucose-stimulated cultured monocytes *in vitro*, different Cr3+ formulations reduced lipid peroxidation and pro-inflammatory cytokine secretion^32^,^33^,^34^. Moreover, in a hypercholesterolemic rabbit model of atherosclerosis, intramuscular administration of chromium chloride (CrCl_3_) lowered serum cholesterol levels and reduced the size of lipid deposits in coronary and aortic vasculature^35^. Despite a favorable response to Cr3+ in vascular disease, the precise effect and mechanisms of the nutraceutical CrP in large vessels in the setting of diabetic atherosclerosis has remained elusive.

The present study provides the first demonstration that orally administered CrP impedes development of atherosclerotic lesions in STZ-induced hyperglycemic ApoE^−/−^ mice, a mouse model of combined atherosclerosis and type 1 diabetes. Our data suggests that inhibition of TSP-1 expression, possibly mediated via reduced protein O-glycosylation, and blockade of VSMC phenotypic switching in the large vessel are important atheroprotective mechanisms of CrP *in vivo*. Notably, we have shown that genetic deletion of TSP-1 protects ApoE^−/−^ mice against hyperglycemia-induced atherosclerosis, mimicking the protective phenotype of CrP-treated diabetic atherosclerotic mice.

## Results

### Oral chromium picolinate has no effect on body weight, blood glucose and lipid profiles in hyperglycemic ApoE^−/−^ mice

**Figure 1:**
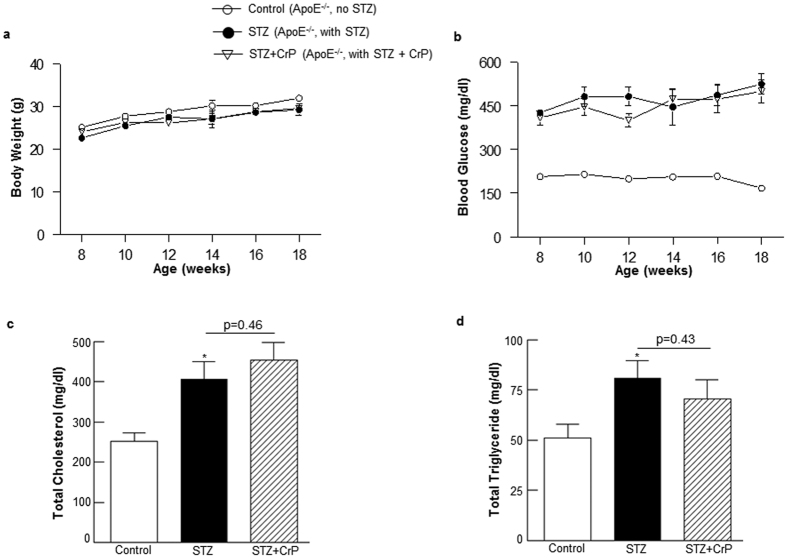
Effect of chromium picolinate *in vivo* on body weight, non-fasted blood glucose, plasma total cholesterol and total triglyceride levels in STZ-induced hyperglycemic ApoE^−/−^ mice. Six weeks old male ApoE^−/−^ mice were treated with 50 mg/Kg/day streptozotocin or sodium citrate buffer (vehicle control) i.p. for 5 consecutive days. This was followed by treatment with or without CrP (8 μg/Kg/day) in drinking water beginning at 8 weeks of age. (**a**) Body weight and (**b**) non-fasted blood glucose levels were measured every two weeks from 8–18 weeks of age. (**c**) Plasma total cholesterol and (**d**) plasma total triglyceride levels were measured at end point of the study (18 weeks). Results are expressed are mean ± SD (n = 10–17 mice per group); *p ≤ 0.05 vs. Control.

No significant differences in body weights were observed in STZ-induced hyperglycemic ApoE^−/−^ mice treated with or without CrP compared with non-hyperglycemic ApoE^−/−^ mice (Fig. 1a). As expected, non-fasted blood glucose levels increased 2–3-fold following STZ treatment in ApoE^−/−^ mice vs. non-STZ-treated ApoE^−/−^ (Fig. 1b); consistent with earlier reports, multiple low-dose STZ did not adversely affect animal well-being or survival. Interestingly, under conditions of experimental type 1 diabetes in ApoE^−/−^ mice, CrP provided in drinking water had no effect on the non-fasted blood glucose levels in these mice. Although a slight reduction (~15%) in glucose levels was noted at 12 weeks of age following CrP administration, this effect was abolished at later time points and the animals continued to remain hyperglycemic attaining non-fasted blood glucose levels ≥250 mg/dl. Moreover, while hyperglycemia increased plasma total cholesterol and total triglyceride levels in STZ-ApoE^−/−^ mice (~1.6-fold versus ApoE^−/−^ control), there was no statistically significant effect of CrP administration on the lipid profiles in hyperglycemic ApoE^−/−^ mice (Figs. 1c,d). Similar to these findings, CrP did not affect either body weight or total cholesterol levels in non-diabetic ApoE^−/−^ control mice (data not shown).

### Chromium picolinate impedes hyperglycemia-induced atherosclerosis in ApoE^−/−^ mice

**Figure 2:**
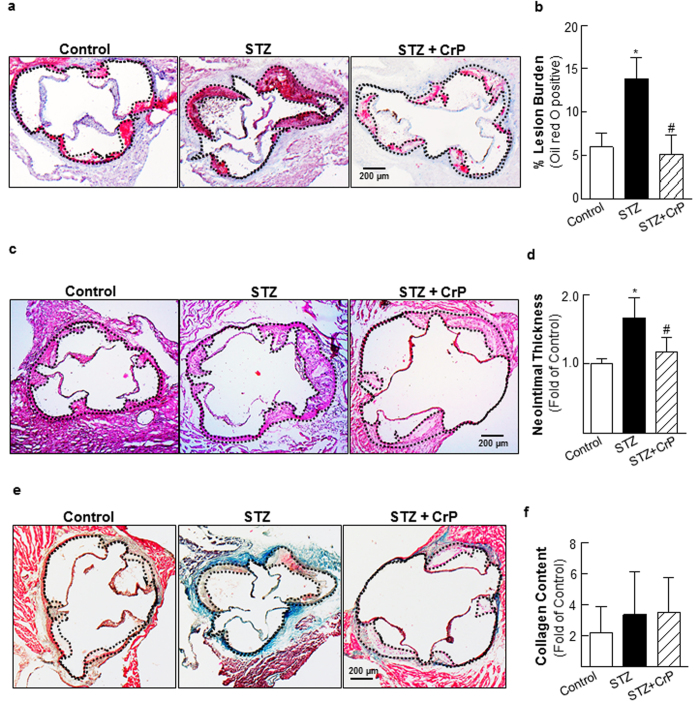
Chromium picolinate *in vivo* attenuates atherosclerotic lesion formation in STZ-induced hyperglycemic ApoE^−/−^ mice. STZ-induced hyperglycemic ApoE^−/−^ mice were treated with or without CrP (8 μg/Kg/day) from 8–18 wks of age. Aortic root morphometry was performed as described in Methods. All comparisons were made between Control (ApoE^−/−^ no STZ), STZ (ApoE^−/−^ with STZ) and STZ + CrP (ApoE^−/−^ with STZ plus CrP). Shown are (**a,b**) representative images and summary data for quantification of ORO-positive staining (Control: n = 6; STZ: n = 7; STZ + CrP: n = 7), (**c,d**) representative H & E images and summary data for neointimal thickness (Control: n = 5; STZ: n = 7; STZ + CrP: n = 7), (**e,f**) representative images and summary data for quantification of MT staining (Control: n = 6; STZ: n = 8; STZ+CrP: n = 8). All microscopic images were captured at 4X magnification. Specific regions used for analyses are marked by dotted lines. Results are presented as fold of Control; all values are expressed as mean ± SD; ^*^p ≤ 0.003 vs. Control, ^#^p ≤ 0.026 vs. STZ.

Atherosclerotic lesion development was examined in all treatment groups using aortic root morphometry. Lipid-filled lesions were significantly increased in STZ-induced hyperglycemic ApoE^−/−^ mice compared with age-matched non-hyperglycemic ApoE^−/−^ littermates, shown by oil red O (ORO) staining. In contrast, CrP treatment diminished lesion formation in hyperglycemic ApoE^−/−^ mice (Fig. 2a). Quantification of ORO-positive staining revealed that while lesion burden was augmented in STZ-ApoE^−/−^ mice (2.3-fold vs. Controls), CrP remarkably reduced lipid-filled lesions in hyperglycemic ApoE^−/−^ (62.6% vs. STZ, Fig. 2b). Aortic root H & E staining showed reduced neointimal thickening in response to CrP (Fig. 2c). Specifically, while the intimal-medial thickness was increased by 66.7% in aortic roots of hyperglycemic ApoE^−/−^ mice compared to non-hyperglycemic ApoE^−/−^, CrP-treated mice demonstrated decreased neointimal thickening (30% vs. STZ only, Fig. 2d). Aortic root morphometry using Masson-Trichrome (MT) staining showed no statistically significant differences in the lesion collagen content in hyperglycemic ApoE^−/−^ mice vs. non-hyperglycemic ApoE^−/−^, although higher collagen amounts were noted in regions outside the aortic root vessel wall in STZ-treated ApoE^−/−^ mice. Moreover, CrP treatment had no effect on collagen accumulation in the aortic root lesions of hyperglycemic ApoE^−/−^ mice compared to STZ only animals (Fig. 2e,f). Interestingly, lesion formation remained unaffected by CrP in ApoE^−/−^ control mice that were not subjected to STZ-induced hyperglycemia (Supplementary Fig. S1).

**Figure 3:**
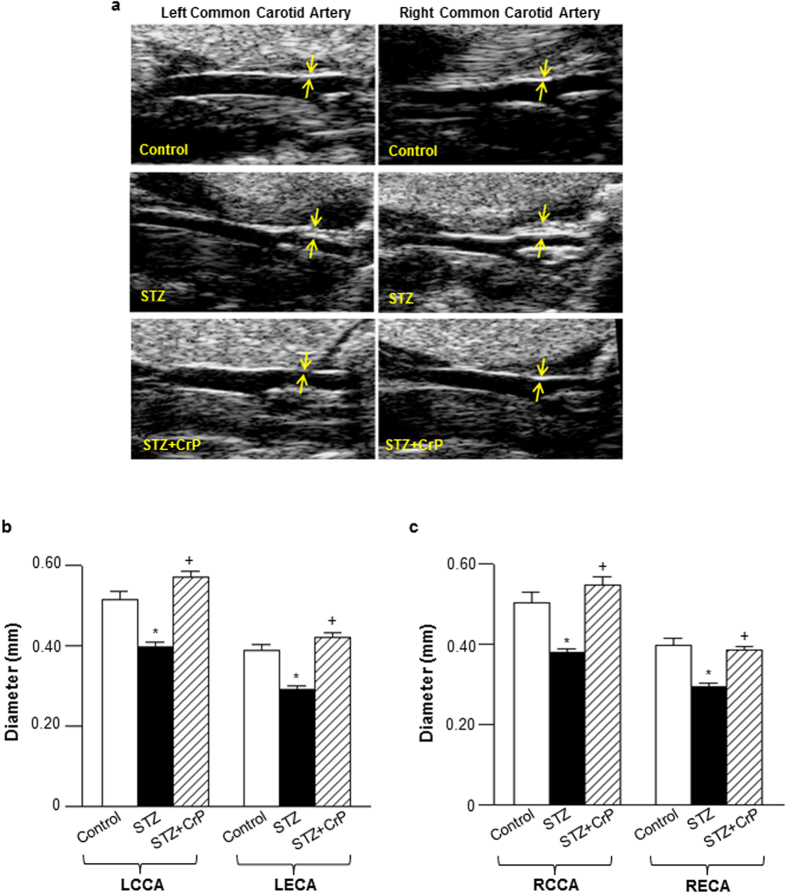
Effect of oral chromium picolinate on lesion formation in carotid vessels measured by High-Frequency Ultrasound Imaging. (**a**) Shown are representative ultrasound images of left and right common carotid artery; yellow arrows indicate neointimal thickening of vessel wall resulting in reduction in vessel diameter in STZ mice. Shown are the summary data for **(b)**, left and **(c)**, right carotid artery diameters. LCCA- left common carotid artery; LECA- left external carotid artery; RCCA- right common carotid artery; RECA- right external carotid artery. Results are expressed as mean ± SD (n = 4–7 mice/group) *p ≤ 0.0001 vs. Control; ^+^p ≤ 0.0001 vs. STZ.

Atherosclerotic lesions were also monitored using a non-invasive auxiliary approach, High-frequency Ultrasound Imaging. Congruent to the aortic root morphometric data, both left ventricular outflow tract and transaortic arch diameters were reduced (~20%) in STZ-ApoE^−/−^ vs. non-STZ-ApoE^−/−^ mice. Notably, this reduction in vessel diameter was completely abrogated in CrP-treated mice (Supplementary Table S1). Furthermore, ultrasound imaging of carotid vessels revealed significant reduction in vessel diameter in hyperglycemic ApoE^−/−^ mice (~25% vs. Control); in contrast, this decrease in vessel diameter was completely obliterated in STZ-ApoE^−/−^ mice treated with CrP (Figs. 3a–c). Further histological experiments were conducted to verify whether the reduction in vessel diameter in STZ-ApoE^−/−^ mice was related to formation of atherosclerotic lesions in the carotid vasculature. Specifically, H & E staining of carotid vessel tissue sections derived from STZ-induced hyperglycemic ApoE^−/−^ mice revealed distinct lesions in the vessel wall resulting in luminal obstruction (Supplemental Fig. S2a). Importantly, a strong negative correlation (-0.91) was noted between the carotid vessel internal diameter and % luminal obstruction of the carotid vessels (Supplemental Fig. S2b), validating the ultrasound assessments of lesion development in the carotid vasculature (Fig. 3). Taken together, these data demonstrate an atheroprotective effect of oral CrP in hyperglycemic ApoE^−/−^ mice.

### Chromium picolinate prevents hyperglycemia-induced increase in cellular proliferation and inflammatory lesion burden in STZ-treated ApoE^−/−^ mice

**Figure 4:**
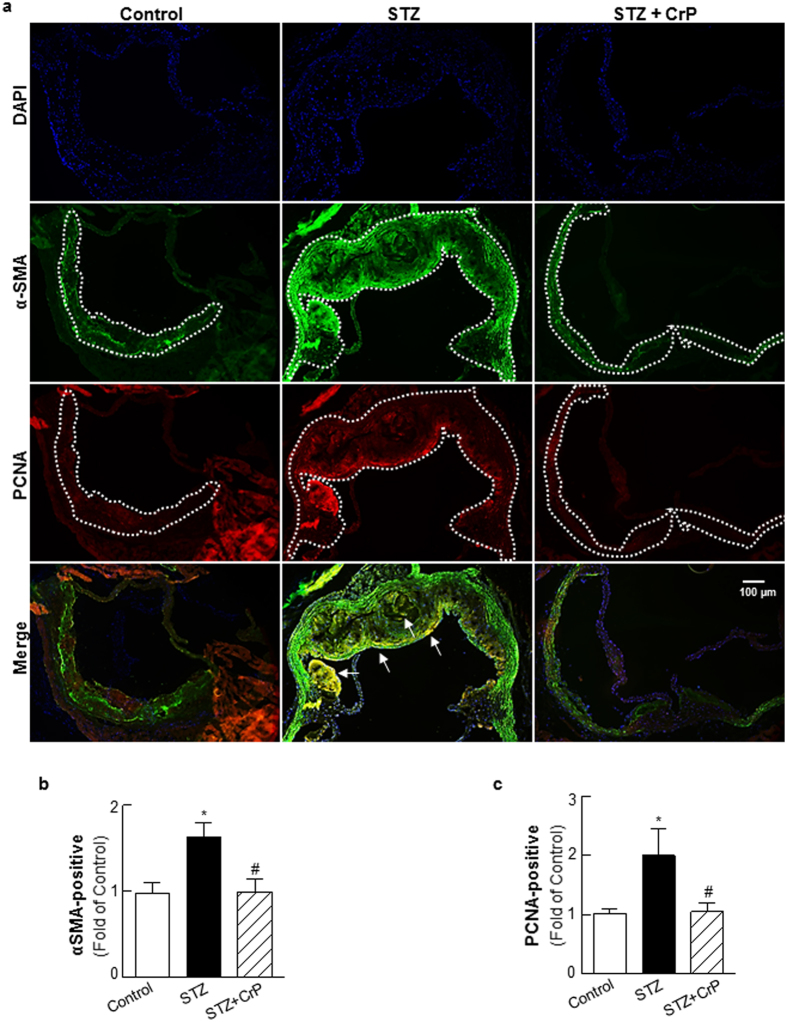
Chromium picolinate *in vivo* prevents hyperglycemia-induced smooth muscle cell abundance and increased cellular proliferation in aortic root lesions of STZ-treated ApoE^−/−^ mice. Shown are (**a**) representative images for PCNA and α-SMA co-staining (10x magnification). Regions used for immunostaining quantifications are outlined by dotted lines; arrows indicate PCNA-positive smooth muscle cells. (**b**,**c**) Summary data for quantification of α-SMA-and PCNA-positive staining. All results are presented as fold of Control and values are expressed as mean ± SD (Control: n = 6; STZ: n = 7; STZ+CrP: n = 6); ^*^p < 0.0001 vs. Control, ^#^p < 0.0001 vs. STZ.

**Figure 5:**
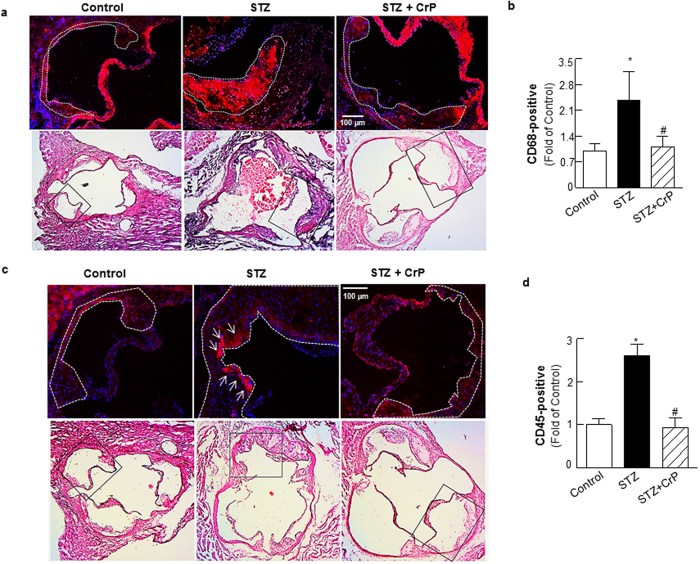
Chromium picolinate *in vivo* prevents hyperglycemia-induced macrophage and leukocyte infiltration into aortic root lesions of STZ-treated ApoE^−/−^ mice. Shown are representative images and summary data for quantification of (**a**,**b**) CD68-positive staining and (**c**,**d**) CD45-positive staining. All images were captured at 15X magnification. Histology of the immunofluorescence images are shown in the corresponding H & E-stained images (indicated by the black box). Specific regions of the immunofluorescence images used for data analyses are marked by dotted lines. Results are presented as fold of Control and all values are expressed are mean ± SD (for CD45, Control: n = 6; STZ: n = 6; STZ+CrP: n = 5; for CD68, Control: n = 5; STZ: n = 5; STZ+CrP: n = 5); ^*^p ≤ 0.0008 vs. Control; ^#^p ≤ 0.0018 vs. STZ.

Next, we characterized lesion cellularity in response to CrP *in vivo*. Smooth muscle cell abundance and cell proliferation was assessed using α-SMA and PCNA immunohistochemistry, respectively. STZ-induced hyperglycemia increased α-SMA (1.6-fold) and PCNA staining (2.0-fold) in ApoE^−/−^ mice, indicating enhanced smooth muscle cell content and cell proliferation. On the contrary, there was a significant decrease in α-SMA and PCNA positive staining in aortic roots of STZ-ApoE^−/−^ mice that received CrP (~40–48% vs. STZ only, Fig. 4); as expected, α-SMA immunostaining demonstrated positive medial staining of smooth muscle cells in control mice whereas increased α-SMA staining was limited to the neointimal layer in the aortic root of STZ mice (Supplemental Fig. S3). The PCNA staining patterns were confirmed using a second proliferation marker, Ki67. As shown in Supplemental Fig. S4a,b, Ki67 expression was increased (2.2-fold) in aortic root lesions of STZ-induced hyperglycemic ApoE^−/−^ mice compared to ApoE^−/−^ control mice, recapitulating the PCNA immunostaining results (Fig. 4c). Moreover, CrP treatment reduced (54%) Ki67 expression similar to its effect on PCNA expression in hyperglycemic ApoE^−/−^ mice compared to STZ only ApoE^−/−^ (Supplemental Fig. S4a,b). Notably, double immunofluorescence experiments using α-SMA and PCNA antibodies demonstrated increased PCNA-positive smooth muscle cell content in aortic root lesions of STZ mice vs. controls. In contrast, increased smooth muscle cell proliferation was prevented in aortic root sections of STZ + CrP mice, depicted by a lack of PCNA-positive α-SMA staining (Fig. 4a, merge). Next, immunohistochemistry demonstrated enhanced macrophage and leukocyte infiltration into aortic root lesions of hyperglycemic ApoE^−/−^ mice while the lesion burden of inflammatory cells was reduced in CrP-treated hyperglycemic mice (Figs. 5a–c). Specifically, immunostaining quantifications revealed > 2.4-fold increase in CD68 and CD45 expression in aortic root lesions of STZ-ApoE^−/−^ mice compared with ApoE^−/−^ controls. In contrast, oral CrP decreased both CD68 and CD45 expression in aortic roots of hyperglycemic ApoE^−/−^ mice (54–64% vs. STZ only, Figs. 5b–d) indicative of attenuated macrophage and leukocyte accumulation, respectively. In each case, the specificity of the immunofluorescence staining was confirmed in parallel aortic root sections incubated in the absence of the corresponding primary antibody (Supplementary Fig. S5). Together, these results demonstrate that CrP *in vivo* prevents hyperglycemia-induced smooth muscle cell and inflammatory cell abundance as well as inhibits smooth muscle cell proliferation in diabetic atherosclerotic mice, resembling a state of reduced plaque development.

### Chromium picolinate *in vivo* inhibits TSP-1 expression, decreases protein O-glycosylation and blocks VSMC phenotypic switching in aortic vessels of STZ-induced hyperglycemic ApoE^−/−^ mice

**Figure 6:**
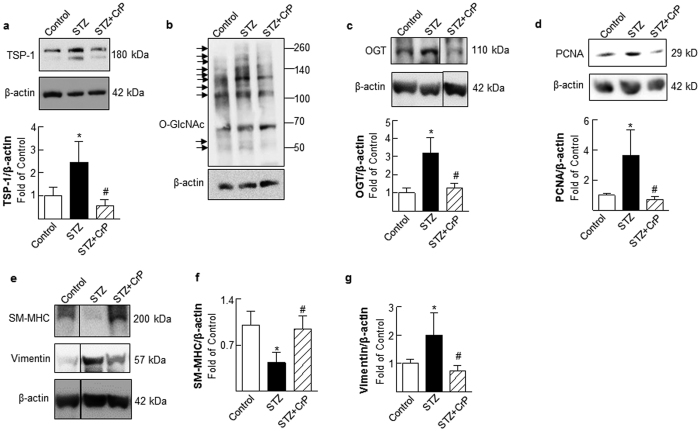
Chromium picolinate *in vivo* inhibits TSP-1, OGT, PCNA and Vimentin expression, increases SM-MHC expression and reduces protein O-glycosylation in aortic lysates of STZ-induced hyperglycemic ApoE^−/−^ mice. Shown are representative immunoblots depicting (**a**) TSP-1, (**b**) O-GlcNAc, (**c**) OGT, (**d**) PCNA, (**e**) SM-MHC expression (upper panel) and vimentin expression (middle panel). Membranes were probed with anti-β-actin used as loading control. In each case, graphs represent summary data for densitometric quantification of immunoblots (at least 3 mice per group). For **c** and **e**, lane images show proteins detected on a single immunoblot; however, lanes were rearranged for clarity of presentation; the corresponding original uncropped blots are presented in Supplementary Fig. S7. All results are presented as fold of Control; values are expressed as means ± SD; ^*^p ≤ 0.05 vs. Control; ^#^p ≤ 0.05 vs. STZ.

We reported earlier that both CrCl_3_ and CrP *in vitro* inhibit TSP-1 expression in glucose-stimulated HASMC primary cultures^19^. To investigate whether the atheroprotective action of CrP observed in the current study correlates with TSP-1 expression *in vivo*, aortic tissue lysates prepared from all treatment groups were subjected to immunoblotting. As shown in Fig. 6a, TSP-1 expression was increased in aortic vessels of hyperglycemic ApoE^−/−^ mice (2.45-fold vs. Control). Concomitant to enhanced TSP-1 expression, hyperglycemia augmented protein O-GlcNAc levels in the aortic vasculature of STZ-ApoE^−/−^ mice. Specifically, immunoblotting of aortic lysates revealed elevated O-GlcNAc levels on several proteins ranging from approximately 100–260 kDa and 45–60 kDa in STZ-ApoE^−/−^ mice (Fig. 6b). This increase in protein O-GlcNAcylation was also accompanied with enhanced OGT expression (3.2-fold, Fig. 6c), a major regulator of O-GlcNAcylation. In contrast, TSP-1 expression was inhibited in aortic vessels of hyperglycemic ApoE^−/−^ treated with CrP (76.7% vs. STZ, Fig. 6a). Moreover, downregulation of TSP-1 expression was accompanied with reduced O-GlcNAc protein modification and attenuated OGT expression in hyperglycemic ApoE^−/−^ mice subjected to CrP treatment (~55% vs. STZ, Fig. 6b,c). Notably, while STZ-induced hyperglycemia augmented cell proliferation marker PCNA expression in ApoE^−/−^ mice (3.6-fold vs. Control), CrP administration significantly inhibited PCNA expression (80% vs. STZ, Fig. 6d). Consistent with effects on cell proliferation, hyperglycemic ApoE^−/−^ mice showed enhanced vimentin expression together with reduced SM-MHC expression in the aortic vasculature. Specifically, densitometry of immunoblots revealed 2-fold increase in vimentin expression and 56.5% decrease in SM-MHC expression in aortic lysates of STZ-ApoE^−/−^ mice compared with ApoE^−/−^ Controls. On the contrary, CrP significantly inhibited vimentin expression (62.5%) while increasing SM-MHC expression (2.18-fold) in hyperglycemic ApoE^−/−^ mice (Figs. 6e–g). Together, these data demonstrate an important link between attenuated lesion formation, downregulation of TSP-1 expression and reduced protein O-GlcNAcylation in CrP- treated hyperglycemic ApoE^−/−^ mice.

### TSP-1 deficiency prevents development of atherosclerotic lesions in STZ-induced hyperglycemic ApoE^−/−^ mice

**Figure 7:**
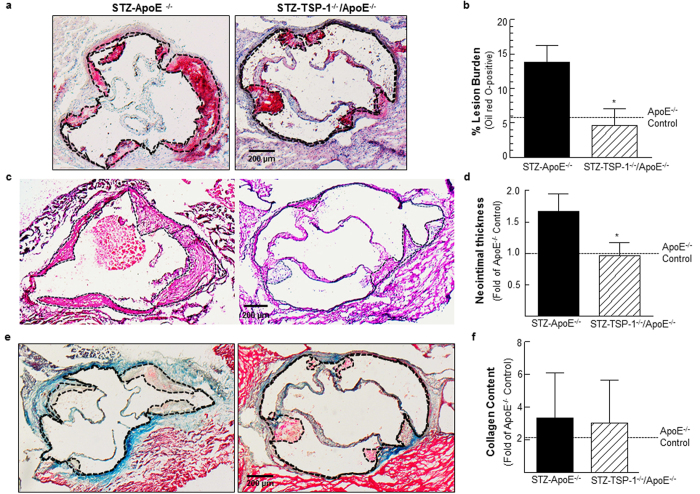
Deletion of TSP-1 prevents formation of atherosclerotic lesions in STZ-induced hyperglycemic ApoE^−/−^ mice. Age-matched male ApoE^−/−^ and TSP-1^−/−^/ApoE^−/−^ dKO mice were subjected to STZ-induced hyperglycemia; aortic root morphometry was performed as described in Methods. Shown are (**a,b**) representative ORO images and summary data for quantification of ORO positive staining (STZ-ApoE: n = 7; STZ-TSP-1/ApoE: n = 5); (**c,d**) representative H & E images and summary data for neointimal thickness (STZ-ApoE: n = 7; STZ-TSP-1/ApoE: n = 5); (**e,f**) representative images and summary data for quantification of MT positive staining (STZ-ApoE: n = 8; STZ-TSP-1/ApoE: n = 5). Specific regions used for analyses are indicated by dotted lines. Results are presented as fold of ApoE^−/−^ Control; all values are expressed as mean ± SD; *p ≤ 0.0013 vs. STZ-ApoE^−/−^.

**Figure 8:**
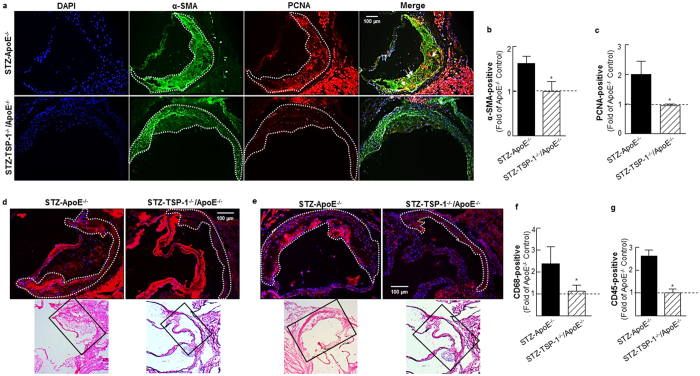
TSP-1 deficiency attenuates cell proliferation, lowers SMC abundance and reduces inflammatory cell content in aortic root lesions of hyperglycemic ApoE^−/−^ mice. Shown are representative images for (**a**) PCNA and α-SMA co-staining (10X magnification), (**b**,**c**) quantification data for PCNA and α-SMA staining (STZ-ApoE^−/−^: n = 7; STZ-TSP-1/ApoE: n = 6), (**d**) CD68 staining (15X magnification) depicting macrophage content and (**e**) CD45 staining (15X magnification) depicting leukocyte abundance and (**f**,**g**) summary data for quantification of CD68 (STZ-ApoE: n = 5; STZ-TSP-1/ApoE: n = 5) and CD45 (STZ-ApoE: n = 6; STZ-TSP-1/ApoE: n = 5) positive staining. Regions used for analysis are marked via dotted lines; arrows indicate PCNA-positive smooth muscle cells. Histology of the immunofluorescence images are shown in the corresponding H & E-stained images (indicated by black box). Results are presented as fold of ApoE^−/−^ Control. All values are expressed are mean ± SD; *p ≤ 0.0019 vs. STZ-ApoE^−/−^.

To investigate a direct role of TSP-1 in hyperglycemia-induced atherosclerosis, we generated TSP-1^−/−^/ApoE^−/−^ double knockout mice that were subjected to STZ-induced hyperglycemia, as described in Methods; these mice were compared with age-matched STZ-induced hyperglycemic ApoE^−/−^ littermates, both genotypes being at 18 weeks of age. Similar to STZ-treated ApoE^−/−^, TSP-1^−/−^/ApoE^−/−^ dKO mice developed significant hyperglycemia upon STZ treatment achieving non-fasted blood glucose levels ≥ 250 mg/dl. However, under these conditions of experimental diabetes, no statistically significant differences were observed in the body weights, plasma total cholesterol and total triglyceride levels between the mice genotypes (Supplementary Table S2). Aortic root morphometry showed remarkable decrease in lipid content (Fig. 7a) and neointimal thickening (Fig. 7c) in hyperglycemic TSP-1^−/−^/ApoE^−/−^ mice compared with age-matched hyperglycemic ApoE^−/−^ littermates. Specifically, morphometric quantification depicted reduced lesion burden (2.9-fold) and neointimal thickness (1.7-fold) in aortic roots of STZ-treated TSP-1^−/−^/ApoE^−/−^ mice (vs. STZ-ApoE^−/−^, Fig. 7b–d). Similar to CrP-treated mice, there was no difference in the collagen content of lesions in either genotypes in response to hyperglycemia (Fig. 7e,f). Furthermore, cellular characterization of lesions revealed attenuated PCNA and α-SMA expression in hyperglycemic TSP-1^−/−^/ApoE^−/−^ dKO mice (vs. hyperglycemic ApoE^−/−^, Fig. 8a–c); while STZ-TSP-1^−/−^/ApoE^−/−^ mice showed positive medial SMA staining, enhanced α-SMA staining was limited to the neointimal surface of the aortic root in STZ-ApoE^−/−^ mice. Importantly, double immunofluorescence staining showed lower abundance of PCNA-positive smooth muscle cells in aortic root lesions of STZ-TSP-1^−/−^/ApoE^−/−^, depicting attenuated smooth muscle cell proliferation (Fig. 8a, merge), compared to STZ-ApoE^−/−^ mice. Further, both CD68 and CD45 expression levels were ameliorated in hyperglycemic TSP-1^−/−^/ApoE^−/−^ dKO mice (vs. STZ-ApoE^−/−^, Figs. 8d–g). Specific immunostaining quantifications showed marked reduction in PCNA expression (2.1-fold), further confirmed by Ki67 immunostaining (Supplemental Fig. S4c,d); moreover, α-SMA, CD68 and CD45 expression profiles were attenuated in aortic root lesions of STZ-induced hyperglycemic TSP-1^−/−^/ApoE^−/−^ mice (1.6-, 2.1- and 2.6-fold, respectively vs. age-matched STZ-ApoE^−/−^ littermates), indicating attenuated cell proliferation, lower smooth muscle cell abundance and reduced macrophage as well as leukocyte lesion invasion. Collectively, these data clearly demonstrate that TSP-1 deletion protects ApoE^−/−^ mice against hyperglycemia-induced atherosclerosis.

## Discussion

The present study provides the first demonstration for an atheroprotective effect of the nutraceutical CrP in a mouse model of diabetic atherosclerosis. We further show that attenuated lesion progression is accompanied with inhibition of TSP-1 expression in response to CrP *in vivo*. Notably, the current work delineates a direct role of TSP-1 in hyperglycemia-driven atherosclerosis.

Clinical data on the cardiovascular benefits of Cr3+ in diabetes have been agnostic^36^,^37^, confounded by the question of whether the effects are mediated via glycemic regulation. Contrary to few earlier reports^26^,^34^,^38^, orally administered CrP did not have any effect on circulating glucose levels in hyperglycemic ApoE^−/−^ mice in the current study. While such discrepancy could be attributed to differences in species (rat vs. mice), Cr3+ formulation and dose as well as animal models (wild-type vs. ApoE^−/−^) utilized, it is important to note that the reduced susceptibility to atherosclerosis in CrP-treated mice was not related to lower lipid profiles in these animals; indeed, plasma total cholesterol and total triglyceride levels were not significantly different between hyperglycemic ApoE^−/−^ mice treated with and without CrP. A possible explanation for such species-dependent differential glycemic response may relate to generation of a metabolite specific to rats, capable of modulating glucose metabolism. As such, our findings suggest an alternate mechanism of atheroprotection by Cr3+, independent of glucose and lipid control.

Previous epidemiological data and animal studies using rabbits have indicated a correlation between Cr3+ intake, incidence of coronary artery disease and serum lipid deposition^31^,^35^,^39^. Both *in vitro* and *in vivo* studies have implicated that Cr3+ may lower risks of vascular inflammation under conditions of elevated glucose levels^32^,^33^,^34^. In line with these reports, the present study has revealed that oral CrP significantly impedes lesion formation and reduces neointimal thickening in the aortic sinus of type 1 diabetic atherosclerotic mice. These morphometric results were also confirmed by ultrasound imaging of the aortic and carotid vasculature. Previous studies have indicated a correlation between conventional ORO staining methods and Ultrasound Imaging approaches for vascular lesion detection in mice^40^. Our data lend additional support to ultrasound imaging as a useful non-invasive methodology for progressive atherosclerotic lesion monitoring in mice. Reduced lesion severity in response to CrP was further illustrated by cellular lesion characterization. Morphometric quantification of aortic root lesions revealed that CrP prevents plaque development in hyperglycemic ApoE^−/−^ mice, depicted by the profound decrease in hyperglycemia-induced proliferative smooth muscle cell abundance as well as macrophage and leukocyte infiltration.

Atherosclerosis is a multifactorial progressive disease comprising of a series of cellular and molecular events, triggering enhanced inflammatory response in the vessel wall. Internalization of atherogenic lipoproteins by monocyte-derived macrophages and activation of inflammatory pathways are important pathogenic mediators of atherosclerosis. This, in turn, may initiate release of a repertoire of growth factors and cytokines promoting VSMC activation, a key step responsible for initiation and progression of atherosclerotic lesions^41^. Diabetic patients are predisposed to aberrant VSMC activation^42^, characterized by enhanced migratory, proliferative and synthetic SMC phenotype. Previous work from our laboratory and others^16^,^17^,^19^ have shown that hyperglycemia, mimicking diabetes, upregulates the proatherogenic protein TSP-1 expression. TSP-1, a secreted glycoprotein, is expressed by many different cell types including endothelial cells, SMC and fibroblasts^16^, and has been widely implicated in atherosclerotic vascular disease^14^,^43^. The multidomain structure of TSP-1 has been attributed to the cell-and tissue-specific mechanisms of TSP-1 upregulation observed under glucose stimulation^17^,^44^,^45^. Earlier reports, including ours, have demonstrated that intracellular protein O-GlcNAcylation, a dynamic posttranslational protein modification, plays a pivotal role in the transcriptional upregulation of TSP-1 in VSMC and endothelial cells^17^,^18^,^44^. More recently, we have shown that both CrCl_3_ and CrP, regardless of its anionic ligand, reduces protein O-GlcNAcylation in primary HASMC cultures; notably reduced O-GlcNAcylation was accompanied with downregulation of TSP-1 expression in glucose-stimulated cells treated with Cr3+^19^. The current data that inhibition of TSP-1 expression occurs concomitant to attenuated OGT and protein O-GlcNAc expression support the overall concept that altered O-GlcNAcylation of nucleocytoplasmic proteins modulates downregulation of TSP-1 expression by CrP in diabetic macrovessels.

Growing evidence indicates that TSP-1 stimulates VSMC migration and proliferation, contributing to neointimal hyperplasia^46^. We have previously shown that high glucose-induced TSP-1 expression leads to abnormally enhanced cell proliferation in HASMC cultures. Specifically, anti-TSP-1 antibody and TSP-1-targeted siRNA blocked glucose-stimulated HASMC proliferation *in vitro*^17^. More recently, we demonstrated that pharmacological concentrations of CrCl_3_ and CrP inhibit TSP-1 expression and attenuate HASMC proliferation in glucose-stimulated cells^19^. Our current findings that CrP *in vivo* abrogates TSP-1 expression together with lowered SMC content and reduced cell proliferation fits well with the role of TSP-1 on VSMC activation. Interestingly, as opposed to Cr3+’s inhibitory effects on glucose-induced proliferation of aortic SMC cultures isolated from wild-type mice, we have found that the anti-proliferative effects of Cr3+ were completely obliterated in glucose-stimulated aortic SMCs derived from TSP-1 transgenic mice, constitutively overexpressing TSP-1 in the arterial SMC (Supplemental Fig. S6); these data extend support to the idea that the anti-proliferative effects of Cr3+ are specific for TSP-1.

In a healthy vessel, SMCs typically localize within medial layers of the arterial wall expressing a plethora of proteins and signaling mediators that modulate its contractile functions. However, exposure to proatherogenic stimuli such as hyperglycemia provokes VSMC phenotypic transition from a quiescent ‘contractile’ state to the proliferative ‘synthetic’ phase^47^, capable of increased lipid uptake and production of extracellular matrix proteins. Our results showing enhanced SM-MHC (SMC contractile marker) expression together with decreased vimentin (SMC synthetic marker) expression in CrP-treated mice suggest a possible regulatory role of Cr3 + on VSMC de-differentiation. Cogent to earlier reports, our data suggest that reduced protein O-GlcNAcylation by CrP may block TSP-1 upregulation in the large vessel impeding enhanced VSMC proliferation and atherosclerotic lesion formation in diabetes. Given that diabetic ApoE^−/−^ mice have elevated cholesterol levels, one might argue that inhibition of atherosclerosis by CrP may be due to changes in the hyperlipidemic status of these animals. However, it is worth noting that the lipid levels remained unaffected by CrP in the current study despite attenuated lesion burden. Future studies are currently underway to determine the conceptual link between O-GlcNAc signaling and VSMC de-differentiation in diabetic atherosclerosis.

Finally, the present study provides the first evidence for a direct role of TSP-1 in hyperglycemia-induced atherosclerosis. Specifically, TSP-1 deficiency diminished both lesion severity and cellularity in STZ-induced type 1 diabetic ApoE^−/−^ mice. Earlier reports have shown that ApoE^−/−^ and TSP-1^−/−^/ApoE^−/−^ dKO mice developed comparable aortic root lesion area and plaque burden following 24 weeks of normocholesterolemic diet^48^. Also, TSP-1^−/−^/ApoE^−/−^ dKO mice at 36 weeks of age manifested increased collagen accumulation and inflammatory cell invasion into lesions triggering enhanced necrotic core formation compared to age-matched ApoE^−/−^ littermates; these results have suggested a potential role of TSP-1 in macrophage-mediated phagocytosis and plaque maturation^48^. As opposed to these earlier findings, we have found reduced macrophage and leukocyte abundance in the aortic root lesions of diabetic TSP-1^−/−^/ApoE^−/−^ dKO mice compared with age-matched diabetic ApoE^−/−^ littermates. Importantly, lack of TSP-1 protected ApoE^−/−^ mice against hyperglycemia-induced atherosclerosis. Moreover, we have shown that hyperglycemic ApoE^−/−^ mice with TSP-1 deficiency mimicked the protective phenotype of CrP-treated diabetic atherosclerotic mice, which may have significant clinical implications.

Interestingly, congruent with earlier reports^48^, no significant difference in lipid-filled lesions was noted between ‘non-diabetic’ ApoE^−/−^ and age-matched ‘non-diabetic’ TSP-1^−/−^/ApoE^−/−^ littermate mice (data not shown). These results clearly highlight a role of TSP-1 that may be specific for hyperglycemia-driven atherosclerosis, with a significant bearing upon diabetic vascular disease. Our data also concur with the counterbalancing effects of TSP-1 on early vs late stage lesions^49^. Reassuringly, the current findings are in agreement with the previously reported involvement of TSP-1 in endothelium activation and infiltration of monocyte-derived macrophages contributing to foam cell formation^48^, and suggest differential patterns of vascular inflammatory burden triggered by hyperglycemia. Overall, the present study prompts us to speculate that inhibition of TSP-1-mediated VSMC phenotypic transition may represent an underlying mechanism of atheroprotection by CrP in diabetes. Although TSP-1 is well known to affect endothelial cell functions, it however remains unclear at this point how CrP affects endothelial cells; additional studies are needed to determine cell-specific effects of CrP on TSP-1 regulation. Furthermore, the impact of VSMC-specific TSP-1 deletion on diabetic atherogenesis warrants future investigation.

In summary, the present study provides strong evidence for an atheroprotective effect of orally administered CrP in a mouse model of type 1 diabetic atherosclerosis. We have also shown that the anti-atherogenic effect of CrP is accompanied with TSP-1 inhibition and reduced VSMC phenotypic transition. Notably, our data underscore TSP-1 as an important driving force for hyperglycemia-induced atherosclerosis. Taken together, the present study suggests key atheroprotective potential of therapeutic TSP-1 inhibition in diabetic macrovascular complications.

## Methods

### Mouse models

All animal procedures were approved by the Institutional Animal Care and Use Committee at Northeast Ohio Medical University in accordance with the NIH guidelines for the Care and Use of Laboratory Animals. Breeder pairs for ApoE^−/−^ mice (stock # 002052) and TSP-1^−/−^ mice (stock # 006141), congenic with C57BL/6 J mice, were originally purchased from The Jackson Laboratories (Bar Harbor, ME, USA) and the mice colonies were expanded and maintained in our animal facility. TSP-1^−/−^/ApoE^−/−^ double knockout (dKO) mice were generated by intercrossing TSP-1^−/−^ mice with ApoE^−/−^ mice, obtained from our in-house breeding colonies. The first generation of offsprings (F1) for TSP-1 and ApoE allele were genotyped and identified as male and female double heterozygous mice; these double heterozygous mice were then bred leading to the second generation of offsprings (F2). From F2, mice identified as TSP-1^+/−^/ApoE^−/−^ were further intercrossed leading to the generation of the double homozygous TSP-1^−/−^/ApoE^−/−^ mice. ApoE^−/−^ and TSP-1^−/−^ genotypes were confirmed by PCR according to established protocols provided by Jackson Laboratories. All animals were housed in a pathogen-free environment and kept on 12:12 h light/dark cycle. All mice were weaned at 4 weeks of age and provided access to regular chow diet ad libitum until 18 weeks of age.

### Induction of hyperglycemia and chromium picolinate administration

Upon weaning, animals were randomly assigned to three groups: ApoE^−/−^, no STZ (Control); ApoE^−/−^, with STZ (STZ) and ApoE^−/−^, with STZ plus CrP (STZ + CrP). Hyperglycemia was induced in six-week old male ApoE^−/−^ mice using a multiple low-dose STZ (Sigma) regimen, as described previously^50^. Briefly, age-matched ApoE^−/−^ littermate mice were injected intraperitoneally with either STZ (50 mg/Kg/day) or sodium citrate buffer (vehicle control) for 5 consecutive days. Ten days after the first STZ injection, blood samples collected by lateral tail incision were used for glucose estimation using a one-touch glucometer. Mice with non-fasted blood glucose levels >250 mg/dl were identified as hyperglycemic. A subset of these hyperglycemic ApoE^−/−^ mice received chromium picolinate (8 μg Cr3+ /Kg/day) provided in drinking water starting at 8 weeks of age. This particular dose of CrP was chosen based on previous rodent studies^23^,^51^. Additionally, this dose approximately equates to an equivalent dose of 560 μg Cr3+ for a 70-kg adult human, representative of commercially available CrP supplements^22^,^52^. Fresh drinking water ± CrP (Nutrition 21, Purchase, NY) was prepared weekly and CrP concentration was adjusted based on the changes in animal weight. Body weight and non-fasted blood glucose levels were monitored every two weeks; animals were harvested at 18 weeks of age.

In a parallel study, six-week old male TSP-1^−/−^/ApoE^−/−^ dKO mice and age-matched ApoE^−/−^ littermates were subjected to STZ-induced hyperglycemia, as described above. Mice with non-fasted blood glucose levels >250 mg/dl were identified as hyperglycemic. Both genotypes were maintained on regular chow diet ad libitum until 18 weeks of age.

### Plasma Lipid Analyses

After an overnight fasting, subsets of mice sacrificed at endpoint were used for estimation of plasma total cholesterol and total triglyceride levels using standard enzymatic kits (Thermo Fisher, Waltham, MA).

### Aortic Root Morphometry

Mice were euthanized using 200 mg/Kg sodium pentobarbital injected intraperitoneally, perfused with PBS followed by formalin, and the heart, ascending aorta including aortic arch and carotid tissue were isolated. Aortic root sections (8–10 micron thickness) of formalin-fixed, OCT-embedded frozen hearts were cut at the point where the aortic valve leaflets were first visible. Care was taken to ensure that serial sections were collected from regions of the aortic root representing about 100–150 microns following the valve leaflet. Additional care was exercised to ensure that all measurements were taken within similar regions of the aortic root among all treatment groups for quantification and comparison. Sections were concurrently stained with 0.5% w/v Oil red O (ORO), hematoxylin and eosin (H & E) and Masson-trichrome (MT) to assess atherosclerotic lesions, intima-media thickness (IMT) and collagen content respectively, as reported earlier^53^. For ORO and MT staining, sections were counterstained with hematoxylin. All sections were mounted with DPX mounting media, observed using Olympus BX40 microscope and images were captured using 4X magnification. For quantitative morphometry, at least 5 animals per treatment group with an average of 20 tissue sections per group were analyzed using Image J software as previously described^54^. Analysis of collagen content was based on the positive MT staining per plaque area, which included both lipid and non-lipid regions; however, lumen area, valve leaflets, vessel walls and regions outside the vessel walls were excluded in these quantifications. Specifically, lesion area was selected using a magnetic lasso tool in Adobe Photoshop; this was copied and pasted into a new image file which was subsequently used for measuring the MT-stained region using Image J software. Line tracings were drawn to mark the luminal perimeter, the inner perimeter and the outer perimeter of the aortic root or carotid vessel cross-sectional image and the corresponding area were determined. Neointimal thickness was determined by subtracting the aortic root luminal perimeter from the aortic root outer perimeter. Percent luminal obstruction of the carotid vessel was calculated as follows: [(Area enclosed by inner perimeter - Area enclosed by luminal perimeter) x100]/Area enclosed by inner perimeter. All image quantifications were performed by team members blinded to the identity of all sections.

### *En-face* atherosclerotic lesion assay

Mice were euthanized, perfused with PBS followed by formalin, and the heart, ascending aorta including aortic arch and carotid tissue were removed under a dissecting microscope. The entire aorta from the heart, including right and left common carotid arteries, extending 10–20 mm after iliac bifurcations were processed for ‘*en-face*’ quantitative atherosclerotic lesion assay. Briefly, aortic and carotid vessels were dissected free of fat and adventitial tissue, opened longitudinally and stained with 0.05% freshly-made Oil red O (ORO) solution. Each stained aortae was then digitally scanned and the percentage of the aorta covered by ORO-positive lipid-filled lesions was determined using Adobe Photoshop, as reported earlier^53^.

### High-frequency Ultrasound Imaging

Vascular lesions were measured non-invasively by High-frequency Ultrasound Imaging using the Vevo 770 High-resolution Imaging System (VisualSonics, Inc. Toronto, Canada), as previously reported^55^. Briefly, both internal and external diameters of left and right common carotids were measured using B-mode (2-dimensional) images. In addition, left ventricular outflow tract (LVOT) and transverse aortic arch diameters were measured.

### Immunohistochemistry

Aortic root sections from each animal were subjected to immunohistochemistry using anti-PCNA (Abcam, Cambridge, MA), anti-αSMA (Sigma, St. Louis, MO), anti-CD68 (Bioss, Woburn, MA), anti-CD45 (Bioss, Woburn, MA) and anti-Ki67 (Abcam, Cambridge, MA) antibodies. Briefly, tissue sections were incubated in ice-cold acetone (5–10 mins) and blocked with 5% donkey or goat serum (90 mins) at room temperature. Following an overnight incubation with primary antibodies (anti-PCNA-1:200; anti-α-SMA-1:200; anti-CD68–1:50; anti-CD45–1:150; anti-Ki67-1:100) at 4 °C, sections were incubated with Alexa Fluor 488 goat anti-mouse (for α-SMA) or Alexa Flour 594 donkey anti-rabbit IgG secondary antibodies (1:1000 or 1:500) and mounted on DAPI-containing mounting media (Vectashield, Vector Laboratories). For co-staining experiments, consecutive slides from serial sections were sequentially stained first with PCNA antibody followed by α-SMA antibody. To control for non-specific staining, identical sections were incubated in the absence of the corresponding primary antibodies, where no background staining was noted. Sections were observed using Olympus fluorescence IX71 microscope (10X or 15X magnification) and images were digitally captured using a set of identical parameters across all sections, specific for each antibody. For immunohistochemistry quantifications, lesion area within the aortic root was outlined, rest of the image cropped away and specific positive staining within lesions was quantified. For each individual treatment group, at least 5 mice with an average of 20 tissue sections per group were utilized for all quantifications. All immunostaining images were quantified in a blinded randomized manner using the Image J software. Results are expressed as fold of control for positive staining.

### Immunoblotting

Aortic tissue lysates were prepared in SDS lysis buffer, as described earlier^56^ and protein content was determined using BCA protein assay. Equal amounts of proteins (35 μg) were resolved on 8% SDS-PAGE and transferred to PVDF membranes. Immunoblotting was performed using anti-TSP-1 (1:500-1:1000, Neomarkers, Freemont, CA), anti-O-GlcNAc (RL2, 1:1000; Abcam, Cambridge, MA), anti-OGT (1:1000, Cell Signaling, Danvers MA), anti-PCNA (1:300, Abcam, Cambridge, MA), anti-SM-MHC (1:2000, Proteintech, Rosemont, IL) and anti-vimentin (1:1000, Cell Signaling, Danvers, MA) antibodies. Membranes were stripped and re-probed with anti-β-actin used as a loading control; equal protein loading of samples was also confirmed by staining the membranes with Ponceau S. All immunoblot images were captured using Protein Simple and densitometric analyses was performed using the Image J software.

### Primary Cultures of Mouse Aortic Smooth Muscle Cell

Primary cultures of mouse aortic smooth muscle cells (aSMC) isolated from TSP-1-transgenic mice, constitutively overexpressing TSP-1 in the arterial SMCs of the aortic vessel, and wild-type mice were kindly provided as a gift by Dr. Olga Stenina Adognravi (Cleveland Clinic, Cleveland, OH). Cells were maintained in complete DMEM/F12 media supplemented with 10% FBS and 1% antibiotics/antimycotic solution. aSMC primary cultures between passages 3–6 were used in all experiments; the contractile phenotype of aSMC was confirmed by α-SMA staining.

### Cell Proliferation Assay

About 5000–7000 mouse aortic smooth muscle cells were plated on 96-well tissue-culture plates in complete DMEM/F12 medium containing 10% FBS. After allowing for an overnight growth, the cells were placed in low glucose (5.5 mM) serum-free DMEM media and further incubated with or without 20 mM glucose in the presence or absence of 100 nM chromium chloride (CrCl_3_) for 72 hours. Cell proliferation was measured at endpoint using the WST-1 cell proliferation reagent (Cayman Chemicals), as reported earlier^19^. Data are represented as % of Control (wild type); all values are expressed as mean ± SD from four independent experiments.

### Statistical Analyses

For all morphometric and immunohistochemistry quantifications, at least 5 mice per treatment group were utilized with an average of 20 tissue sections per treatment group for each measurement. Sections derived from identical regions of the aortic root following the valve leaflet were used in all treatment groups for quantifications and comparisons. Please note, immunohistochemistry and morphometric data collected from all animals were included in our quantifications. All images were quantified by team members blinded to the identity of the treatment groups, in order to minimize bias and intentional exclusion of animals from the study. Differences in group sizes for some measurements were due to lack of additional tissue sections from the corresponding mice. For immunoblotting, aortic tissue lysates prepared from at least 3 mice per group were utilized. Image J software was used for densitometry of immunoblots and positive staining quantification. For indicated immunoblots, lane images show proteins detected on a single blot; however, lanes were rearranged for clarity of presentation. All data are presented as fold of control; values are expressed as mean ± SD, to depict variability of data. For comparison between two treatment groups, statistical analysis was done using unpaired Student’s t-test. For comparison between three groups, one-way analysis of variance (ANOVA) was used. Differences between mean values were considered statistically significant at P ≤ 0.05.

## Additional Information

**How to cite this article**: Ganguly, R. *et al*. Oral chromium picolinate impedes hyperglycemia-induced atherosclerosis and inhibits proatherogenic protein TSP-1 expression in STZ-induced type 1 diabetic ApoE^−/−^ mice. *Sci. Rep.* **7**, 45279; doi: 10.1038/srep45279 (2017).

**Publisher's note:** Springer Nature remains neutral with regard to jurisdictional claims in published maps and institutional affiliations.

## Supplementary Material

Supplemental Material
