# Supplementary material for: Oral chromium picolinate impedes hyperglycemia-induced atherosclerosis and inhibits proatherogenic protein TSP-1 expression in STZ-induced type 1 diabetic ApoE−/− mice
Source: Sci Rep. 2017 Mar 27;7:45279. doi: 10.1038/srep45279 (PMC5366888; doi:10.1038/srep45279)
Supplement: Supplemental Material [file srep45279-s1.pdf]

SUPPLEMENTAL MATERIAL

Oral chromium picolinate impedes hyperglycemia-induced atherosclerosis and inhibits proatherogenic protein TSP-1 expression in STZ-induced type 1 diabetic ApoE$^{-/-}$ mice

Rituparna Ganguly[+1,2], Soumyadip Sahu[1,2], Vahagn Ohanyan[1], Rebecca Haney[1], Ronaldo J. Chavez[1], Shivani Shah[1], Siri Yalamanchili[1], Priya Raman[1,2 *]

[1]Department of Integrative Medical Sciences, Northeast Ohio Medical University, 4209 State Route 44, Rootstown, OH 44272, USA,

[2]School of Biomedical Sciences, Kent State University, Kent, OH 44240, USA,

*** Corresponding author**: Priya Raman, Department of Integrative Medical Sciences, Northeast Ohio Medical University (NEOMED), 4209 State Route 44, Rootstown, OH 44272-0095, USA. Phone: (330)-325-6425. Fax: (330)-325-5912. Email: praman@neomed.edu

Supplemental Figure S1. Chromium picolinate in vivo has no effect on atherosclerotic lesion formation in 'non-diabetic' ApoE$^{-/-}$ mice. Age-matched male ApoE$^{-/-}$ mice maintained on regular chow diet were treated with or without chromium picolinate (CrP, 8μg/Kg/day) provided in the drinking water from 8-18 wks of age. Shown are (**a**) representative '*en-face*' atherosclerotic lesion images of aortic vessels stained with Oil Red O and (**b**) summary data for Oil Red O-positive staining quantification (n=5-6 mice per group).

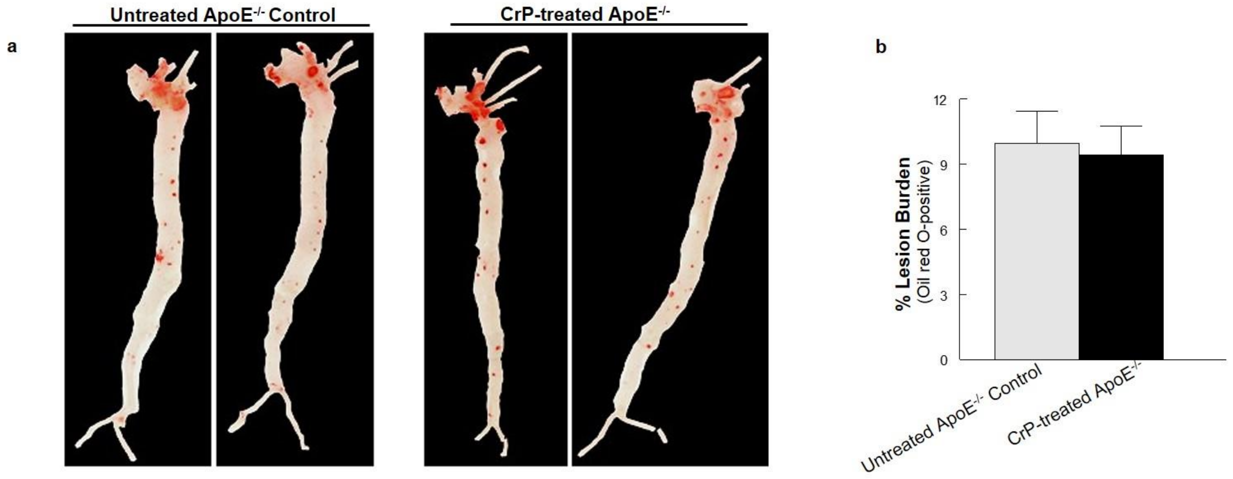

Supplemental Figure S2. H & E staining of carotid vessel tissue sections derived from STZ-ApoE$^{-/-}$ mice was performed as described in Methods. Shown are (**a**) representative H & E-stained image depicting atherosclerotic lesion and (**b**) a scatter plot depicting the correlation between the carotid vessel internal diameter and % luminal obstruction in the carotid vessel. Five different sets of carotid vessels isolated from STZ-ApoE$^{-/-}$ mice were used in these measurements; for histology, 6-10 sections per each individual carotid vessel were utilized; values are expressed as mean ± SD.

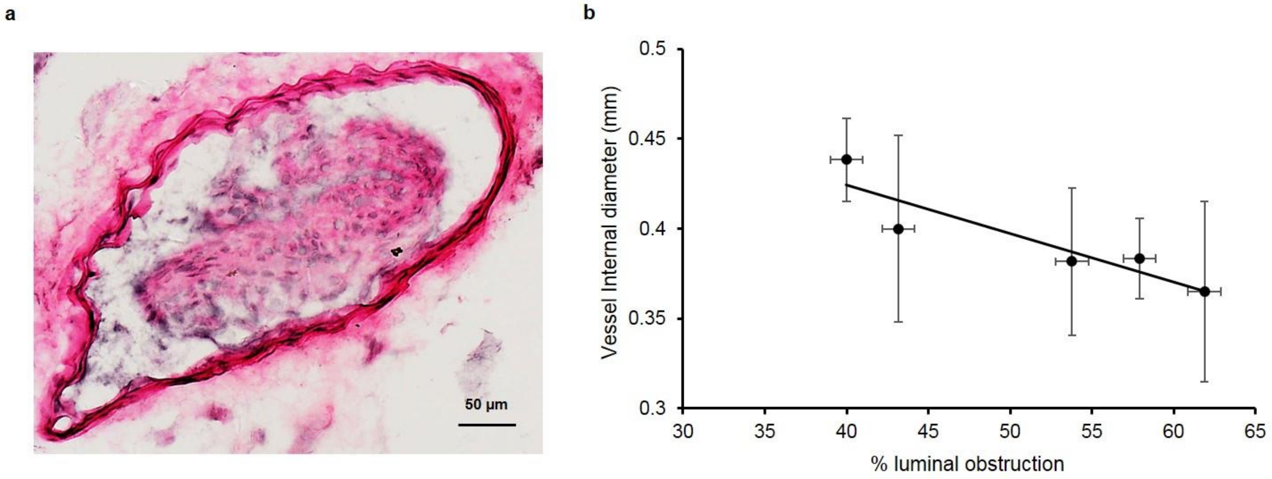

Supplemental Figure S3. Shown are representative images for α-SMA staining of aortic root sections derived from Control (untreated ApoE$^{-/-}$) and STZ-ApoE$^{-/-}$ mice. Note the positive medial staining of smooth muscle cells in Control compared to increased SMA staining in the neointimal layer in STZ mice. m denotes medial and n denotes neointimal layer.

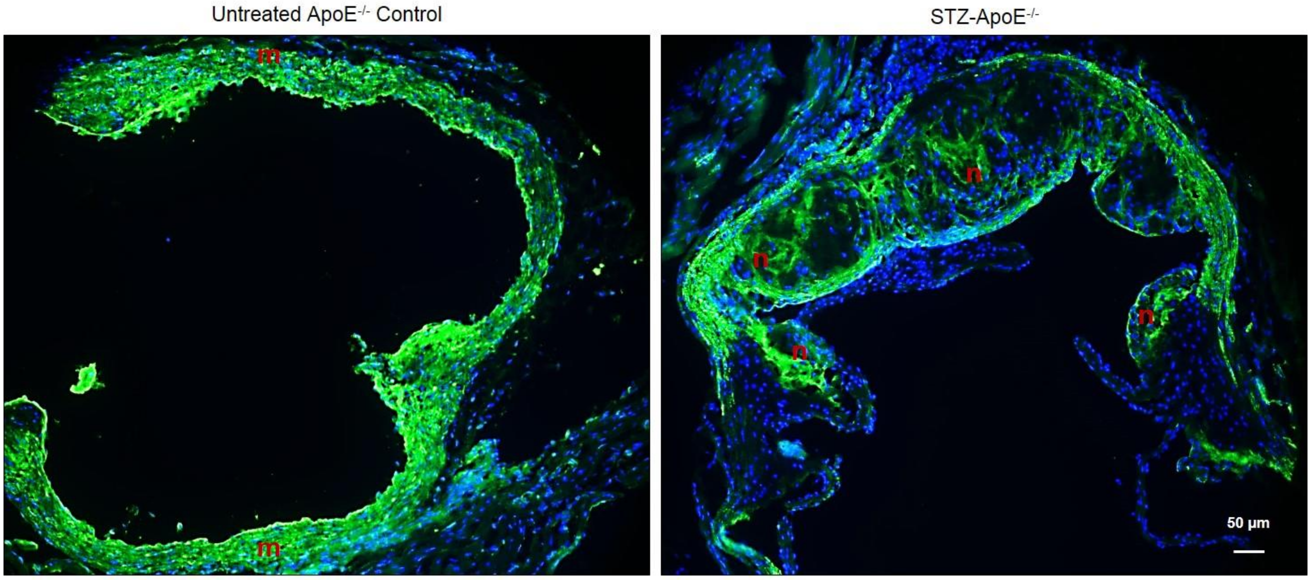

Supplemental Figure S4. Aortic root sections derived from ApoE$^{-/-}$ Control, STZ-ApoE$^{-/-}$, STZ-ApoE$^{-/-}$+CrP and STZ-TSP-1$^{-/-}$/ApoE$^{-/-}$ mice were used in Ki67 immunofluorescence staining as described in Methods. Shown are the representative images and summary data for Ki67 positive staining. All images were captured at 10X magnification. Results are presented as fold of ApoE$^{-/-}$ Control; all values are expressed as mean ± SD (n=5 mice per group); $^{*}p \leq 0.05$; $^{\#}p \leq 0.05$; arrows denote Ki67-stained nuclei.

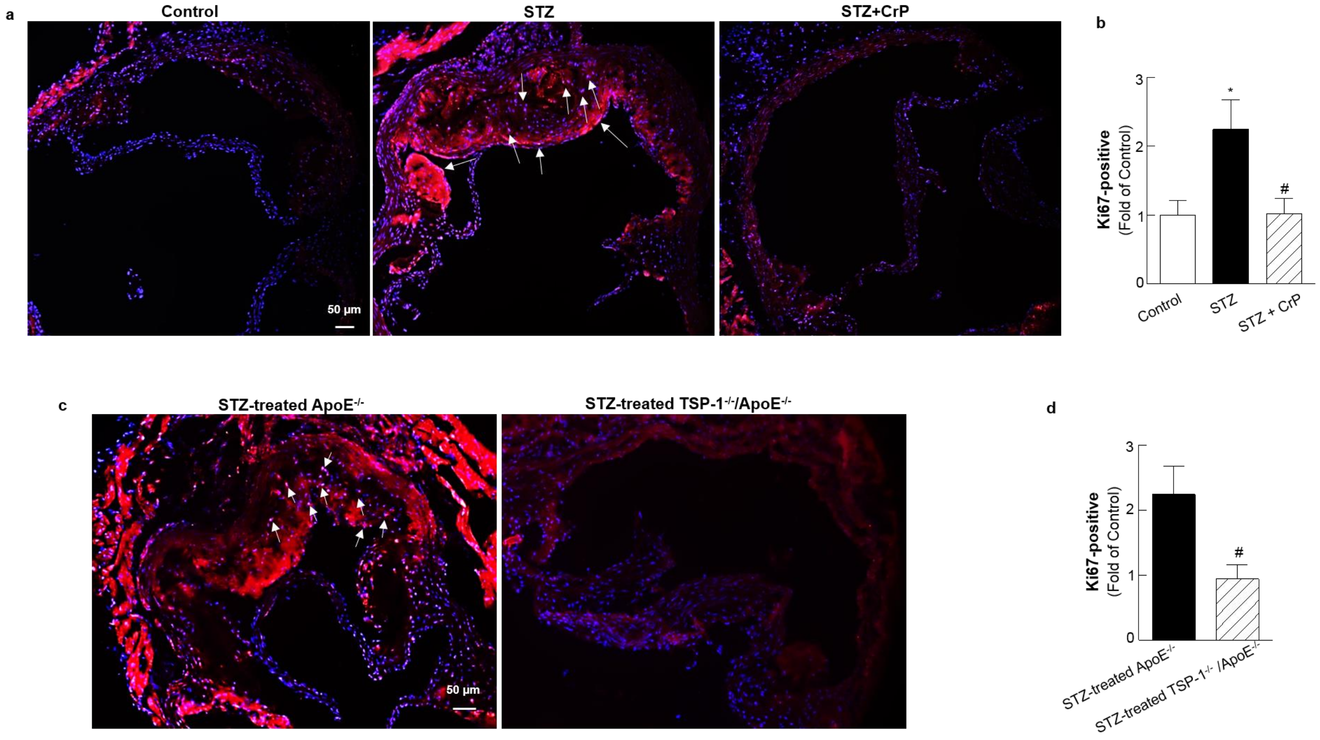

Supplemental Figure S5. Shown are representative negative control images for (**a**) α-SMA and PCNA, (**b**) CD68 and (**c**) CD45 immunofluorescence staining. To control for non-specific staining, identical sections derived from similar regions of the aortic root of STZ-treated $ApoE^{-/-}$ mice were incubated in the presence or absence of the corresponding primary antibodies; briefly, following an overnight incubation with or without relevant primary antibodies at 4^oC, sections were incubated with Alexa Fluor 488 goat anti-mouse (for α-SMA) or Alexa Flour 594 donkey anti-rabbit IgG secondary antibodies for 1 hour. Sections were then mounted on DAPI-containing mounting media. All images were digitally captured using fluorescence microscope at 10X or 15X magnification, as indicated in Methods. In each case, histology of the immunofluorescence images are shown in the corresponding H & E-stained images; dotted lines depict lesion area.

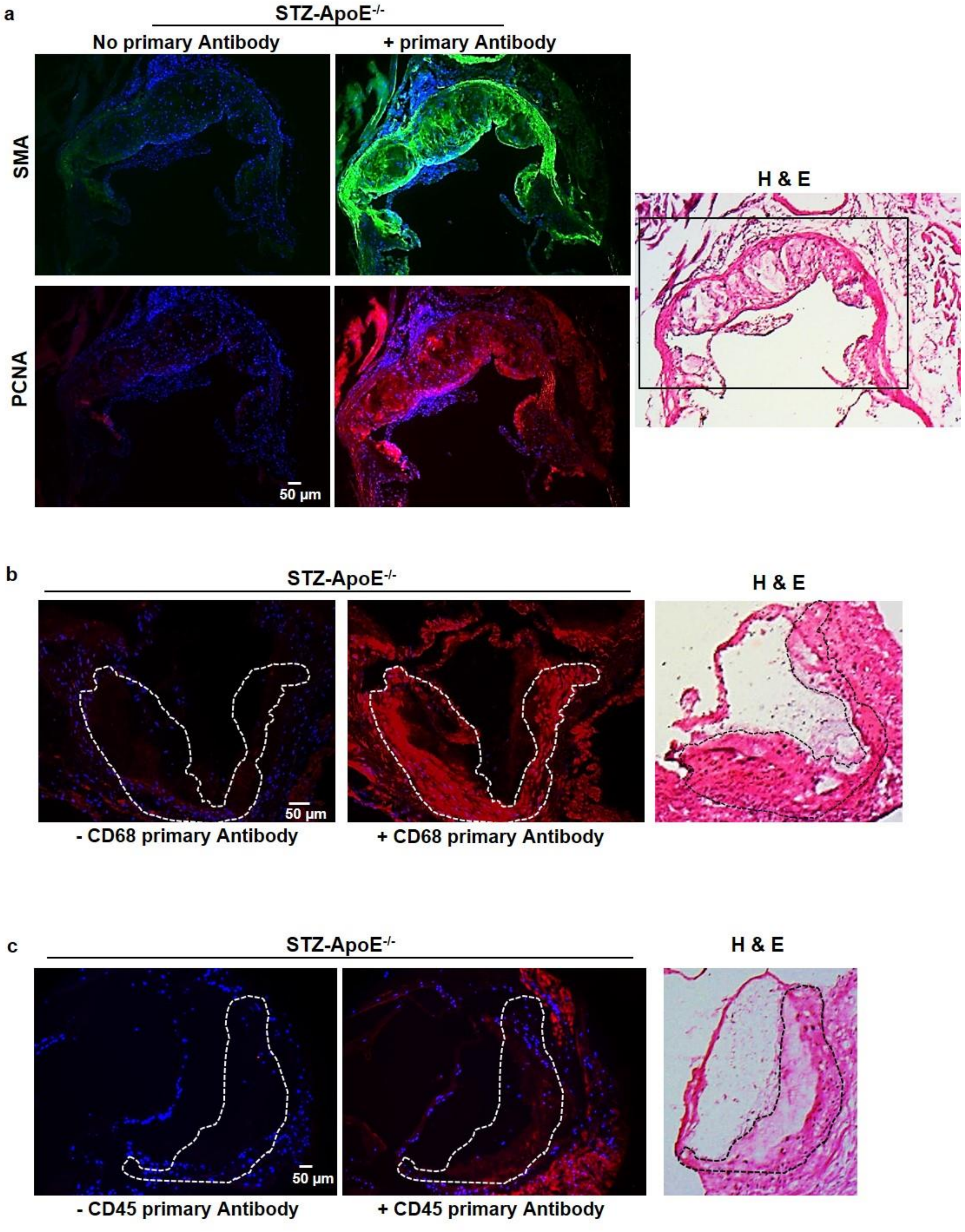
a
STZ-ApoE-/-
No primary Antibody
+ primary Antibody
SMA
PCNA
50 µm
H & E
b
STZ-ApoE-/-
H & E
50 µm
- CD68 primary Antibody
+ CD68 primary Antibody
c
STZ-ApoE-/-
H & E
50 µm
- CD45 primary Antibody
+ CD45 primary Antibody

Supplemental Figure S6. Anti-proliferative effect of chromium is attenuated in TSP-1 transgenic (TSP1-Tg) aortic SMC compared with wild-type aortic SMC. Aortic SMC primary cultures from wild-type (WT) and TSP-1-transgenic (TSP-1-Tg) mice were plated in complete DMEM/F12 media. After an overnight growth, cells were placed in serum-free low glucose DMEM and incubated with or without 20 mM glucose in the presence or absence of 100 nM $CrCl_3$ for 72 hours. Cell proliferation was determined at end point using WST-1 cell proliferation reagent. *$p \leq 0.05$ vs. control; $^{+}p \leq 0.05$ vs. wild-type (Glucose 20mM). Results are expressed as % of Control (wild-type); values represent mean ± SD from 4 independent experiments.

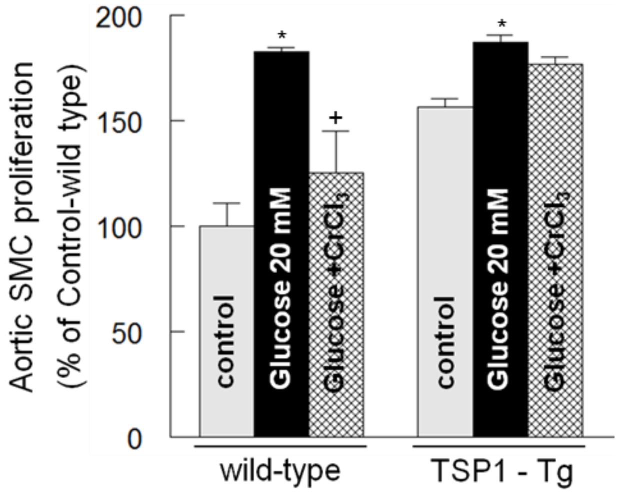

Supplemental Figure S7. Shown are the original uncropped blots for the western blot images shown in Figs. 6c and 6e. For Fig 6c, membrane was first probed with anti-SM-MHC (upper panel) antibody; this was followed by stripping and re-probing of the membrane with anti-vimentin (middle panel) followed by anti-β-actin (lower panel) antibodies. For Fig. 6b, membrane was first probed with anti-OGT antibody; this was followed by stripping and re-probing of the membrane with anti-β-actin antibody. In each case, the individual lanes are marked 1-7. Arrows indicate the position of the relevant proteins.

a)

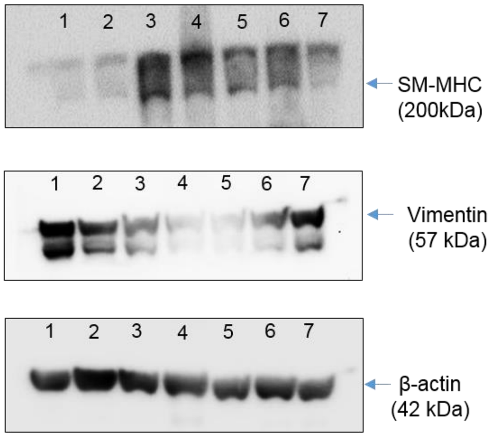

Lane 2: STZ
Lane 3: STZ+CrP
Lane 4: Control

b)

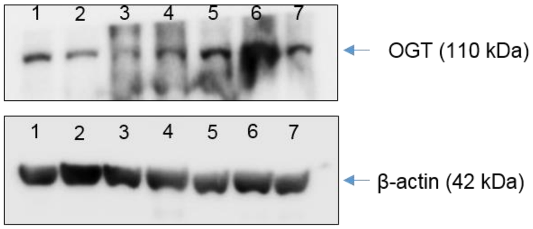

Lane 3: STZ+CrP
Lane 4: Control
Lane 5: STZ

Supplemental Table S1. Chromium picolinate *in vivo* prevents reduction in aortic vessel diameter in STZ-induced hyperglycemic ApoE$^{-/-}$ mice. Left ventricular outflow tract (LVOT) and transaortic arch internal diameters were measured using B-mode ultrasound images obtained from age-matched ApoE$^{-/-}$, STZ-ApoE$^{-/-}$ and CrP-treated STZ-ApoE$^{-/-}$ mice at 18 weeks of age. Values represent mean ± SD; $^{*}p \leq 0.0001$ vs. ApoE$^{-/-}$; $^{\#}p \leq 0.0001$ vs. STZ-ApoE$^{-/-}$.

**Diameter (mm)**	**ApoE$^{-/-}$**	**STZ-ApoE$^{-/-}$**	**CrP-treated STZ-ApoE$^{-/-}$**
Left Ventricular Outflow Tract (LVOT)	1.43 ± 0.07 (n=5)	1.16 ± 0.03* (n=7)	1.46 ± 0.07$^{\#}$ (n=7)
Transaortic Arch	1.46 ± 0.11 (n=3)	1.17 ± 0.06* (n=6)	1.49 ± 0.08$^{\#}$ (n=4)

Supplemental Table S2. Effect of TSP-1 deletion on body weight, non-fasted blood glucose and plasma lipid levels in STZ-treated ApoE$^{-/-}$ mice. Six weeks old male ApoE$^{-/-}$ mice and age-matched TSP-1$^{-/-}$/ApoE$^{-/-}$ littermates were injected i.p with 50mg/Kg/day STZ for 5 consecutive days. Animals were kept on regular chow diet until 18 weeks of age. Body weight and non-fasted blood glucose levels were monitored in both mice genotypes every two weeks and plasma total cholesterol and total triglyceride levels were measured at end point of the study (18 weeks); shown are data collected at endpoint. All results are presented as mean ± SD; STZ-treated ApoE$^{-/-}$ (n=10); STZ-treated TSP-1$^{-/-}$/ApoE$^{-/-}$ (n=8).

	**STZ-treated ApoE$^{-/-}$ (18 wks age)**	**STZ-treated TSP-1$^{-/-}$/ApoE$^{-/-}$ (18 wks age)**
Body weight (g)	29.28 ± 4.15	25.22 ± 3.94
Blood Glucose (mg/dl)	525.17 ± 85.9	600 ± 10.00
Total Cholesterol (mg/dl)	405.37 ± 180.5	427.62 ± 239.3
Total Triglyceride (mg/dl)	80.82 ± 39.6	82.44 ± 58.2
